# Efficacy of administrative intervention for neurosurgical patients with off-label use of alprostadil lipid microsphere

**DOI:** 10.1038/s41598-022-19717-0

**Published:** 2022-09-13

**Authors:** Yuling Luo, Qingze Fan, Yongqi Yu, Lunhui Zhang, Limei Dong, Hongli Luo

**Affiliations:** 1 Department of Pharmacy, The Affiliated Hospital of Southwest Medical University, Luzhou, 646000 Sichuan China; 2 School of Pharmacy, Southwest Medical University, Luzhou, 646000 Sichuan China

**Keywords:** Diseases, Health care, Medical research, Neurology

## Abstract

As an adjuvant drug, alprostadil lipid microsphere injection (Lipo-PGE_1_) is one of the best-selling drugs in China in recent years. However, the off-label use of Lipo-PGE_1_ is very common. This study aimed to investigate the use of Lipo-PGE_1_ and evaluate the clinical effects and economic benefits after administrative intervention on inappropriate use of Lipo-PGE_1_ in neurosurgical patients in a Chinese tertiary hospital. Administrative interventions were implemented from January to December 2018 by reducing the procurement volume of Lipo-PGE_1_, judging the rationality of medical records, and establishing reward and punishment mechanisms. Administrative interventions significantly decreased prescription rate (49.98% vs 22.49%), utilization (22,311 DDDs vs 8334 DDDs), drug use density (43.52 DDDs/TID vs 15.84 DDDs/TID), total expenditure (3.58 million *RMB* vs 1.30 million *RMB*), and average expenditure (2025.04 *RMB* vs 1466.49 *RMB*) of Lipo-PGE_1_. To our delight, these intervention effects were maintained or even better in the 1-year post-intervention period. Moreover, in the intervention and post-intervention phases, the Lipo-PGE_1_ use for no indications as well as inappropriate drug dose, frequency, menstruum type, combination, and contraindication were markedly reduced. Besides, the mean costs (*P* < 0.001), and mean duration (*P* < 0.001) of Lipo-PGE_1_ were also obviously decreased. The administrative intervention obviously reduced the off-label use of Lipo-PGE_1_. However, there still remains a number of inappropriate uses of Lipo-PGE_1_. To further improve the rational use of Lipo-PGE_1_, combination of administrative intervention and real-time clinical pharmacists intervention should be implemented.

## Introduction

Alprostadil is a stable prostaglandin E1 analogue with anti-platelet aggregation, peripheral vascular smooth muscle relaxation, anti-oxidation, endothelium protection, and liver cell membrane stabilization activities^1–5^. Based on this, alprostadil has been used successfully for the treatment of critical leg ischemia, angiospastic disorders, Raynaud’s phenomenon, diabetic peripheral neuropathy, diabetic foot, liver transplantation, etc. by intravenous or intraarterial administration^6–10^. Unfortunately, the conventional alprostadil formulation is rapidly metabolized, resulting in an extremely short half-life of 3–5 min; moreover, its dosage is high in clinical practice thus frequently inducing adverse events. To address these problems, the alprostadil liposome microsphere injection (Lipo-PGE_1_) was originally produced by Mitsubishi Pharmaceutical Co., Ltd. in Tanabe, Japan. Lipo-PGE_1_ could accumulate in inflammatory lesions and diseased vessels due to the enhanced permeability and retention (EPR) effect of lipid microspheres, which significantly reduces the dose of alprostadil and thus minimizes toxic side effects^11–13^. For example, when Lipo-PGE_1_ was used to improve microcirculation, its dosage was only a few tenths of the dosage of conventional formulation, while the drug concentration in the lesions could reach up to 10–20 times than that of conventional formulation. Lipo-PGE_1_ also can control the drug release and prolong drug retention time in vivo to 12–24 hours^14^.

In China, the first generic Lipo-PGE_1_ became commercially available in 1998. Subsequently, more than ten generic Lipo-PGE_1_s were put on the market, including injection and dried emulsion for injection. As an adjuvant drug, the Lipo-PGE_1_ has been reported to be used widely in the clinic for 871 kinds of diseases, including cerebral infarction, diabetes, chronic nephropathy, essential hypertension, sudden deafness, fractures, intervertebral disc disease, chronic obstructive pulmonary disease, etc^15^. However, in China, the summary of product characteristics of generic Lipo-PGE_1_ contains only 4 indications, namely limb ulcer caused by chronic arterial occlusion disease (thromboangiitis obliterans, occlusive arterial sclerosis, etc.) and resting limb pain caused by tiny blood circulation obstacle, improvement of microcirculation of heart head blood-vessel, antithrombotic therapy after organ transplantation, arterial catheter dependence of congenital heart disease, and aid in the treatment of chronic hepatitis. The term “off-label” use refers to use of a drug that is not included in the package insert (approved labeling) for that drug. In this case, the prescriptions for off-label indications use are common in clinical practice. In addition to this, the inappropriate dose, menstruum, administration route, and contraindications are also frequently found in prescriptions.

Currently, the phenomenon of non-indication or off-label drug use is common all over the world^16–20^. Eleven countries have regulations on the off-label drug use, namely America, England, Germany, Italy, Netherland, Australia, New Zealand, South Africa, India, Japan, and China^21^. Accordingly, off-label drug use has its rationality and necessity. In 2015, the “off-label drug use expert consensus” was formed to protect patients and avoid the risk for hospitals and healthcare workers in China. Meanwhile, the Health and Family Planning Commissions of Beijing, Guangdong, Yunnan, and Sichuan Provinces listed Lipo-PGE_1_ in the catalogue of adjuvant drugs regulation. Despite these expert consensuses, regulatory policies, and local enforcement regulations, the use of Lipo-PGE_1_ is still far from optimal. A study showed that 65.41% of patients received Lipo-PGE_1_ treatment with off-label indications, 99.85% of patients used drug with an unreasonable dosage, and 0.39% of patients with contraindications to drug^22^. Another study revealed that off-label Lipo-PGE_1_ use was found in 25 out of 36 clinical departments in a tertiary hospital in China^23^.

Analogously, the inappropriate use of Lipo-PGE_1_ is ubiquitous in the Affiliated Hospital of Southwest Medical University, located in Luzhou, China. There are two kinds of Lipo-PGE_1_s produced by different manufacturers in the hospital, namely alprostadil injection (10 μg, 93 *RMB*) and alprostadil dried emulsion for injection (5 μg, 79 *RMB*). Our previous investigation demonstrated that the sales of Lipo-PGE_1_ (88.75% alprostadil dried emulsion for injection and 11.25% alprostadil injection) ranked first among thousands of drugs in the hospital, and from 2014 to 2017, the prescription rate of Lipo-PGE_1_ for inpatients in the hospital increased annually, which were 4.25%, 10.05%, 12.53%, and 13.63%, respectively. In 2017, the top three departments in the prescription rate of Lipo-PGE_1_ were cardiovasology, infectious gastroenterology, and neurosurgery. It should be noted that, from 2015 to 2017, the prescription rates of neurosurgery were 28.82% (3351/11,627), 44.54% (6068/13,623), and 49.97% (9148/18,307), respectively, which had been at the forefront of 45 departments. Moreover, for Lipo-PGE_1_ treatment, most patients in cardiovasology and infectious gastroenterology may have indications, while most patients in neurosurgery may have no indications, and some patients, instead, may have contraindications (intracranial hemorrhage).

The above problems have drawn great attention from the Hospital Pharmacy Administration and Therapeutics Committee (HPATC), and a range of interventions have been implemented to address these issues. Our previous study had confirmed that the real-time interventions of clinical pharmacist promoted the rational use of prophylactic acid suppressants and resulted in favorable economic outcomes^24^. However, the number of clinical pharmacists is seriously insufficient due to the increase of clinical beds, so an administrative intervention was implemented here for neurosurgery to decrease the inappropriate uses and the costs of Lipo-PGE_1_ from January 1, 2018 to December 31, 2018. Importantly, to the best of our knowledge, there is no related report. The purpose of this self-controlled study was to evaluate the clinical and economic impacts of administrative intervention and thus promote the rational use of Lipo-PGE_1_ in neurosurgical patients in a Chinese tertiary teaching hospital.

## Methods

### Study design

A single-center self-controlled study was performed on inpatients in the Department of Neurosurgery of the Affiliated Hospital of Southwest Medical University, a 4200-bed major academic tertiary hospital with a daily average admission rate of about 5000 patients and more than 130,000 inpatients annually in Luzhou, China. In order to evaluate the efficacy of administrative intervention on off-label use of Lipo-PGE_1_, this study includes three phases, namely: pre-intervention (12 months), intervention (12 months), and post-intervention (12 months). All inpatients that received Lipo-PGE1 and without systemic diseases during hospitalization in the neurosurgery were enrolled. The systemic diseases involve multiple organs and multiple parts of the same tissue, including AIDS, systemic lupus erythematosus, polychondritis, rheumatoid, etc. Subsequently, we used the simple random sampling technique to define the study sample. 300 samples were randomly selected using computer-generated random digits from each intervention stage (2017–2019). Patients were excluded if they were minors (< 18 years old), or were hospitalized for less than 3 days or more than 30 days; patients were also excluded if drugs, improving the microcirculation, were used two weeks prior to the episode of admission, with or without an indication was documented in the medical chart; patients transferring from other medical departments or transferring to other medical departments for further treatment were excluded. In order to evaluate the intervention effect, three outcomes were analyzed in our present study. The first outcome was the use of Lipo-PGE_1_, including the amount and DDDs. The second outcome was the expenditure of Lipo-PGE_1_ from 2017 to 2019. And the last outcome was the rationality of Lipo-PGE_1_ use, which was obtained by the collection of medical records. The rationalities of Lipo-PGE_1_ use in respect of indication, alprostadil selection, dosage, frequency, menstruum, administration route, and combination were analyzed depending on drug instructions and related disease diagnosis and treatment guidelines (Table 1)^25,26^.

**Table 1:** Internal guideline for the rationality of Lipo-PGE_1_ use in the Affiliated Hospital of Southwest Medical University.

Parameter	Justification for rational use
Indications	Treatment of limb ulcer caused by chronic arterial occlusion (thromboangiitis obliterans, arteriosclerosis obliterans, etc.) and limb resting pain caused by microvascular circulation disorder, and improvement of cardiovascular microcirculation disorders
	Antithrombotic therapy after organ transplantation
	Congenital heart disease with ductus arteriosus dependence
	Adjuvant therapy for chronic hepatitis
Agents	Alprostadil injection, alprostadil dried emulsion for injection
Dosage	5–10 μg
Frequency	Quaque die
Menstruum	10 ml sodium chloride injection or 10 ml glucose injection
Route	Intravenous slowly or intravenous drip slowly through a small pot
Contraindications	Severe heart failure (cardiac insufficiency)
	Pregnancy or possible pregnancy
	Allergy to this preparation

### Interventions

Several administrative interventions in the rational use of Lipo-PGE_1_ introduced by the HPATC were performed from January 1, 2018 to December 31, 2018. Administrative intervention refers to the use of economic, legal, policy, and other means by administrative organs to regulate the operation and relationship of hospital pharmaceutical management, so as to ensure the sustained, coordinated, and healthy development of the hospital economy, politics, culture, and other aspects. Such as the establishment of drug management and reward and punishment system, limiting the number of drugs, and other measures. The interventions consisted: (1) Limited prescription authority. Doctors in the departments of cardiology, vascular surgery, and hepatology are authorized to prescribe the drug, while other doctors can only use the drug after consultation in the above three departments; (2) Limited supply of drugs. The pharmacist collected and ranked sales of all drugs in the whole hospital once a month based on the total expenditure during the past month, and then sent feedback to HPATC. If an adjuvant drug was in the top 20 of sales, its purchase would be decreased by 20% in the next month; (3) Evaluation of rationality. The pharmacist randomly selected 10 medical records of patients who received Lipo-PGE_1_ from the top five departments of Lipo-PGE_1_ prescription rates every month and judged the rationality of Lipo-PGE_1_ use according to the criteria. The results were advertised in the Hospital Information System (HIS) and informed to the leadership of clinical departments at the monthly meeting held by the hospital. Then leadership of the departments would fully communicate the results to every clinician; (4) The reward and punishment mechanism was established. The departments and individuals with reasonable and standardized drug use will be praised in the monthly regular meeting. Conversely, the departments and individuals with unreasonable drug use will be criticized in the whole hospital and interviewed by the Discipline Inspection Commission of hospital; in addition, the costs of unreasonable drug use would be deducted from the incentive performance of responsible doctors.

### Data collection and analysis

The HIS, Electronic Medical Records (EMR), and Prescription Automatic Screening System (PASS) of Sichuan Medico Software Research and Development Co. Ltd. were used to collect the numbers of patients, demographic information of patients (sex, age, medical history, diagnosis, and allergies), surgical procedures (name and date), Lipo-PGE_1_ usage (generic name, dosage form, summary of product characteristics, unit price, dose, frequency, solvent, administration route, duration, combinations, and replacement) and cost (total charges for the hospitalization and Lipo-PGE_1_). The data analysis was conducted by another pharmacist who was blinded to the allocation status of patients. All data collected was anonymous and could not be traced back to an individual.

The prescription rate of Lipo-PGE_1_, defined as daily doses (DDDs), DDDs/1000 inpatients per day (DDDs/TID) was used for measuring drug utilization, which was in line with international recommendations. DDDs is defined as “the average maintenance dose per day for a drug used for its main indication in adults”. Since the DDDs of Lipo-PGE_1_ was not recorded in WHO ACT/DDD, DDDs of Lipo-PGE_1_ was identified as 10 μg according to instructions in this study. The Chinese Yuan Renminbi (*RMB*) was used to determine the expenditure of Lipo-PGE_1_ over time. In order to calculate the actual changes during this period, inflation or deflation were not considered. 6.5 *RMB* equals to 1 *US dollar*.

Data were entered and analyzed subsequently by SPSS 22.0. To assess the significant differences among the three stages, a chi-square test was used for the analysis of categorical variables, and one-way ANOVA was used for the analysis of continuous variables. A *P* value less than 0.05 was considered statistically significant.

### Ethical considerations

The presented study was approved by the Clinical Trial Ethics Committee of The Affiliated Hospital of Southwest Medical University (No. KY2022023). All methods were performed following the relevant guidelines and regulations. All patients provided written, informed consent for study participation.

## Results

### Use and expenditure (***RMB***) of Lipo-PGE_1_

The use and expenditure of Lipo-PGE_1_ from 2017 to 2019 were outlined in Table 2. After intervention, the prescription rate of Lipo-PGE_1_ decreased from 49.98% (pre-intervention) to 22.49% (intervention) and 20.87% (post-intervention), respectively, and the utilization and use density of Lipo-PGE_1_ were significantly lower than that of pre-intervention (22,311 DDDs [pre-intervention] vs 8334 DDDs [intervention] vs 6471 DDDs [post-intervention]; 43.52 DDDs/TID [pre-intervention] vs 15.84 DDDs/TID [intervention] vs 11.79 DDDs/TID [post-intervention]). Importantly, decreases in total and average expenditures of Lipo-PGE_1_ were observed (3.58 million *RMB* and 2025.04 *RMB* [pre-intervention] vs 1.30 million *RMB* and 1466.49 *RMB* [intervention] vs 1.02 million *RMB* and 1307.51 *RMB* [post-intervention]). Besides, the percentage of Lipo-PGE_1_ expenditures in total drug expenditures decreased from 8.54% (pre-intervention) to 3.42% (intervention) and 2.50% (post-intervention).

**Table 2:** The use and expenditure (RMB) of Lipo-PGE_1_ for all neurosurgical patients in pre-, during, and post-intervention periods.

Characteristics	Pre-intervention	Intervention	Post-intervention
Inpatients (n)	3530	3909	3762
Mean hospitalization days	14.52	13.46	14.59
**Inpatients with Lipo-PGE**_**1**_** use (n)**^**a**^			
Alprostadil dried emulsion for injection (5 μg)	1761	870	780
Alprostadil injection (10 μg)	31	51	5
**Prescription rate of Lipo-PGE**_**1**_** (%)**^**a**^			
Alprostadil dried emulsion for injection (5 μg)	49.89	22.26	20.73
Alprostadil injection (10 μg)	0.88	1.30	0.13
**Use of Lipo-PGE**_**1**_** (DDDs)**			
Alprostadil dried emulsion for injection (5 μg)	22,201	8064	6446
Alprostadil injection(10 μg)	110	270	25
**Use density of Lipo-PGE**_**1**_			
Alprostadil dried emulsion for injection (5 μg)	43.31	15.33	11.74
Alprostadil injection (10 μg)	0.21	0.51	0.05
Total expenditures of all drugs (million *RMB*)	41.88	38.00	40.96
**Expenditures of Lipo-PGE**_**1**_** (*RMB*)**			
Alprostadil dried emulsion for injection (5 μg)	3,566,096.34	1,275,843.45	1,019,855.05
Alprostadil injection (10 μg)	10,325.62	25,317.90	2325.50
Percentage of Lipo-PGE_1_ expenditures (%)	8.54	3.42	2.50
Average expenditures of all drugs (*RMB*)^b^	11,864.02	9721.16	10,887.47
**Average expenditures of Lipo-PGE**_**1**_** (*RMB*)**^**c**^			
Alprostadil dried emulsion for injection (5 μg)	2025.04	1466.49	1307.51
Alprostadil injection (10 μg)	333.08	496.43	465.10

^a^28 and 42 patients received successively two formulations of Lipo-PGE_1_ before and after the intervention, respectively.

^b^All patients.

^c^Patients with Lipo-PGE_1_ use.

### Characteristics of neurosurgical patients treated with Lipo-PGE_1_ in pre-, during, and post-intervention groups

A total of 1764, 879, and 785 neurosurgical patients were treated with Lipo-PGE_1_ in pre-, during, and post-intervention groups, respectively. As shown in Table 3, the majority of patients were men, and most were 46–65 years of age or older. Alprostadil was wildly used in neurosurgery and was used for more than 20 kinds of clinical diseases in total. Before the intervention of Lipo-PGE_1_ use, the top three ranked diseases were cerebral hemorrhage (28.40%), intracranial space-occupying lesions (11.28%), and cerebral contusion (11.05%); during the intervention, the top three listed diseases were cerebral hemorrhage (28.64%), intracranial injury (12.05%), and cerebral infarction (8.86%); after the intervention, the top three ranked diseases were cerebral infarction (33.76%), cerebral hemorrhage (16.43%), and fracture (7.90%).

**Table 3:** Characteristics of neurosurgery patients treated with Lipo-PGE_1_ in pre-, during, and post-intervention periods.

Characteristics	Pre-intervention	Intervention	Post-intervention
**Sex**			
Female (n, %)	666 (37.76)	310 (35.23)	284 (36.18)
Male (n, %)	1098 (62.24)	570 (64.77)	501 (63.82)
**Age**			
< 18 years	235 (13.32)	25 (2.84)	10 (1.27)
18–45 years	370 (20.98)	145 (16.48)	153 (19.49)
46–65 years	746 (42.29)	478 (54.32)	385 (49.05)
66–85 years	392 (22.22)	218 (24.77)	230 (29.30)
> 85 years	21 (1.19)	14 (1.59)	7 (0.89)
**ICD-10 diagnosis category**			
Cerebral hemorrhage	501 (28.40)	252 (28.64)	129 (16.43)
Intracranial space occupying lesions	199 (11.28)	70 (7.95)	29 (3.69)
Cerebral contusion	195 (11.05)	73 (8.30)	40 (5.10)
Intracranial injury	158 (8.96)	106 (12.05)	33 (4.20)
Fracture (skull, thoracic or lumbar vertebra)	152 (8.62)	59 (6.70)	62 (7.90)
Subdural or epidural hematoma	128 (7.26)	62 (7.05)	59 (7.52)
Intracranial aneurysm	86 (4.88)	24 (2.73)	15 (1.91)
Cerebral infarction	96 (5.44)	78 (8.86)	265 (33.76)
Protrusion of cervical or lumbar intervertebral disc	40 (2.27)	30 (3.41)	21 (2.68)
Hydrocephalus	37 (2.10)	25 (2.84)	57 (7.26)
Epilepsy	20 (1.13)	10 (1.14)	22 (2.80)
Others	152 (8.62)	91 (10.34)	53 (6.75)

### General characteristics of patients and expenditure of Lipo-PGE_1_ in pre-, during, and post-intervention periods

**Figure 1:**
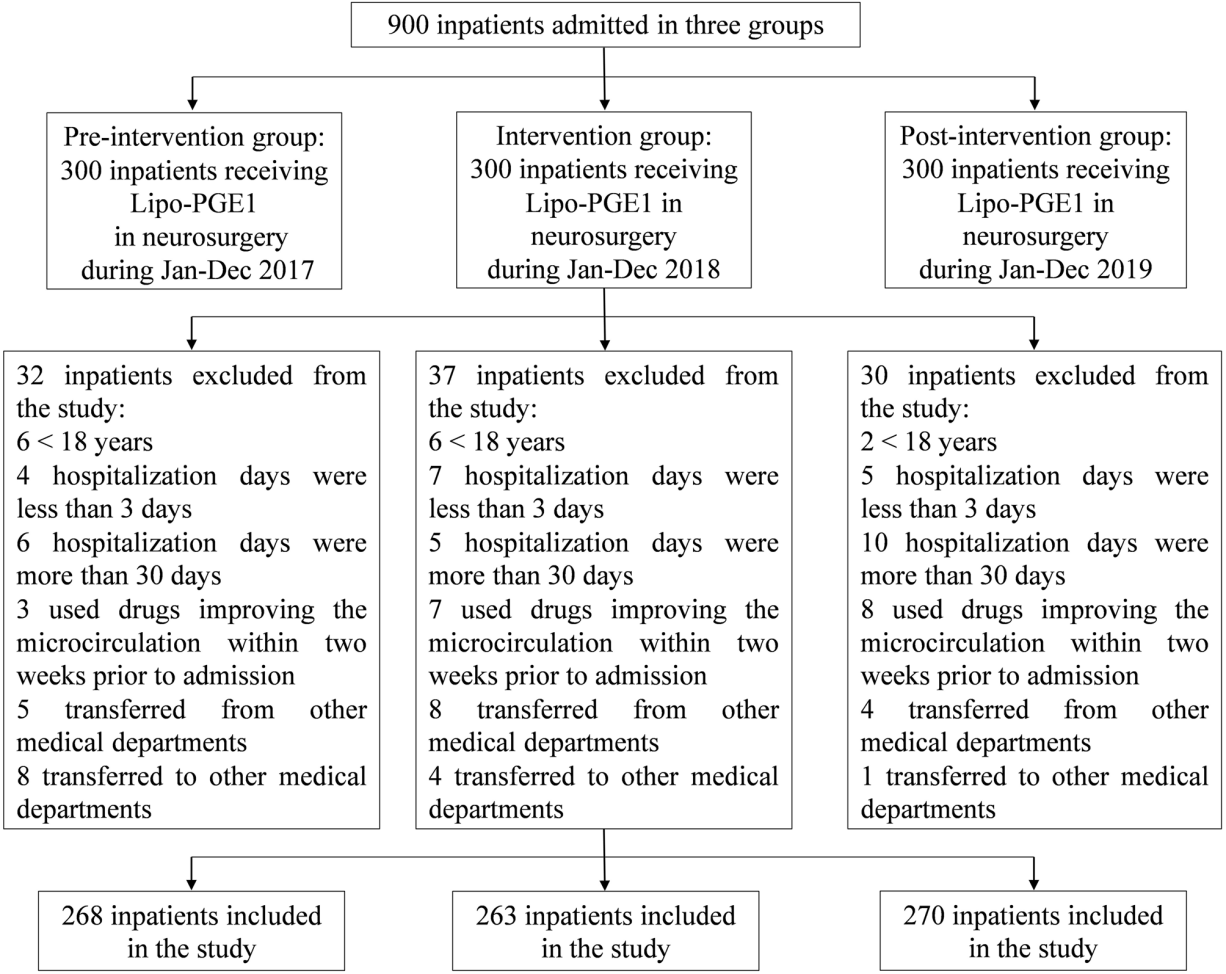
Patient selection and flow chart.

In the beginning, a total of 900 patients were enrolled in this study. Patients were randomly divided into three groups with 300 patients per group. Then, we excluded 99 patients who did not meet our inclusion criteria. At last, 801 patients in total were included for further research with 268 in the pre-intervention group, 263 in the intervention group, and 270 in the post-intervention group (Fig. 1). The general characteristics of the patients in three groups were listed in Table 4. The three groups were similar in demographics and clinical characteristics, such as sex, age, and operation. There was no change in the price of Lipo-PGE_1_, other drugs, and hospital service during the study period. There were no significant differences in mean overall hospitalization costs and mean hospitalization days among three groups (*P* > 0.05). However, significant reductions in mean Lipo-PGE_1_ costs (*P* < 0.001) and mean duration of Lipo-PGE_1_ (*P* < 0.001) were observed in the intervention group. Moreover, these reductions were maintained in the post-intervention group.

**Table 4:** General characteristics of patients and expenditure of Lipo-PGE_1_ in pre-, during, and post-intervention periods.

Characteristics	Pre-intervention (n = 268)	Intervention (n = 263)	Post-intervention (n = 270)	*p*-value^a^	*p*-value^b^
Male (n, %)	169 (63.06)	171 (65.02)	179 (66.30)	NS	NS
Age, mean ± SD	57.91 ± 14.46	59.35 ± 16.48	56.02 ± 15.38	NS	0.039
Operation (n, %)	84 (31.34)	79 (30.04)	90 (33.33)	NS	NS
Mean overall hospitalization costs (*RMB*)	32,256.20	28,652.58	32,870.29	NS	NS
Mean Lipo-PGE_1_ costs (*RMB*)	1714.77	1097.32	1224.79	< 0.001	NS
Mean duration of Lipo-PGE_1_ (day)	10.53	7.01	7.75	< 0.001	NS
Mean hospitalization days	14.19	13.44	14.56	NS	NS

^a^The statistical differences between pre-intervention and intervention groups.

^b^The statistical differences between intervention and post-intervention groups.

*NS* not significant (*P* > 0.05).

### Frequency of Lipo-PGE_1_ usage

During the study period, some patients may take two types of Lipo-PGE_1_ due to the drug shortage, which caused the frequency of Lipo-PGE_1_ usage to be higher than the number of patients. As shown in Table 5, the frequency of Lipo-PGE_1_ usage was 273 in the pre-intervention group and 280 in the intervention group, which increased the mean usage of Lipo-PGE_1_. The prescription rates of alprostadil dried emulsion for injection and alprostadil injection were 98.17% and 1.83% in the pre-intervention group, 93.21% and 6.79% in the intervention group, and 100% and zero in the post-intervention group, respectively.

**Table 5:** The use of Lipo-PGE_1_ in pre-, during, and post-intervention periods.

Characteristics	Pre-intervention (n = 268)	Intervention (n = 263)	Post-intervention (n = 270)
Frequency of Lipo-PGE_1_ usage	273	280	270
Alprostadil dried emulsion for injection (n, %)	268 (98.17)	261 (93.21)	270 (100.00)
Alprostadil injection (n, %)	5 (1.83)	19 (6.79)	0 (0.00)
Mean usage of Lipo-PGE_1_	1.02	1.07	1

### Rate of inappropriate Lipo-PGE_1_ use

According to the established criteria, inappropriate Lipo-PGE_1_ uses in the pre-, during, and post-intervention groups were listed in Table 6. As expected, a clear reduction was observed in the no indication for Lipo-PGE_1_ use and the cases of inappropriate drug dose, frequency, menstruum type, combination, and contraindications in the intervention group, compared to the pre-intervention group (*P* < 0.001). Moreover, these intervention effects were maintained or even better in the post-intervention group. 15 patients used Lipo-PGE_1_ at a dose of 20 μg qd, and 16 patients used 10 μg q12h before intervention. 65 patients were treated with Lipo-PGE_1_ under the inappropriate diluting menstruum, such as invert sugar injection, multiple electrolytes, and invert sugar injection. Besides, this drug was used with an inappropriate volume of menstruum and an inappropriate administration route (intravenous drip) in all the pre-, during, and post-intervention groups, so the percentage of patients who met all the seven criteria was zero in three groups. As shown in Table 7, the volume of menstruum was 50 mL or 100 mL or 250 mL or 500 mL.

**Table 6:** Rate of inappropriate Lipo-PGE_1_ use in pre-, during, and post-intervention periods.

Characteristics	Pre-intervention (n, %)	Intervention (n, %)	Post-intervention (n, %)	*p*-value
No indication	248 (92.54)	229 (87.07)	170 (62.96%)	< 0.001
Inappropriate dose	31 (11.57)	0 (0.00)	0 (0.00)	< 0.001
Inappropriate frequency	16 (5.97)	0 (0.00)	0 (0.00)	< 0.001
Inappropriate menstruum type	65 (24.25)	0 (0.00)	0 (0.00)	< 0.001
Inappropriate volume of menstruum	268 (100)	263 (100)	270 (100)	NS
Inappropriat administration route	268 (100)	263 (100)	270 (100)	NS
Unnecessary combination	82 (30.60)	25 (9.51)	20 (7.41)	< 0.001
Contraindications	31 (11.57)	10 (3.80)	11 (4.07)	< 0.001

*NS* not significant (*P* > 0.05).

**Table 7:** Menstruum of Lipo-PGE_1_ in pre-, during, and post-intervention periods.

Menstruum	Pre-intervention (n, %)	Intervention (n, %)	Post-intervention (n, %)
0.9% Sodium chloride injection 50 mL	0 (0.00)	6 (2.28)	1 (0.37)
0.9% Sodium chloride injection 100 mL	177 (66.04)	252 (95.82)	242 (89.63)
0.9% Sodium chloride injection 250 mL	21 (7.84)	5 (1.90)	27 (10.00)
5% Glucose injection 100 mL	5 (1.87)	0 (0.00)	0 (0.00)
Invert sugar injection 250 mL	43 (16.04)	0 (0.00)	0 (0.00)
Multiple electrolytes and invert sugar injection 250 mL	11 (4.10)	0 (0.00)	0 (0.00)
Multiple electrolytes and invert sugar injection 500 mL	11 (4.10)	0 (0.00)	0 (0.00)

## Discussion

To our knowledge, we report for the first time the improvement of rational use of Lipo-PGE_1_ and the saving of medication costs after administrative intervention in neurosurgical patients. As expected, the simple administrative intervention significantly declined the prescription rate, use, and expenditure of Lipo-PGE_1_. To our delight, these reductions were maintained or even lower in the 1-year post-intervention period. However, the rationality of Lipo-PGE_1_ use was not satisfactory in the intervention and post-intervention phases, where none of these medical records was entirely reasonable according to the established criteria.

In the present study, we found that Lipo-PGE_1_ was widely used in neurosurgery. However, only 12.93% and 38.04% of patients had an indication in the intervention and post-intervention stages, respectively, which was similar to other recently published studies^22,27^. At the same time, no indication for Lipo-PGE_1_ use was common in many hospitals in China^15^. Based on our results, we propose that there are three main reasons for the overuse of Lipo-PGE_1_ in hospitals in China. First, a large number of studies have reported that the application of Lipo-PGE_1_ in the treatment of a variety of diseases leads to the blind preference of clinicians, but the literature quality is poor. Second, some misconceptions about Lipo-PGE1 are frequent among surgeons and they believe that when Lipo-PGE_1_ is administered in the short-term, no side effects are observed in addition to intravenous inflammation. Finally, some surgeons overuse Lipo-PGE_1_ for commercial purposes due to the high price.

Meanwhile, in our study we found that 11.57%, 3.8%, and 4.07% of the cases had contraindications in pre-, during, and post-intervention groups, respectively. Moreover, some patients took Lipo-PGE_1_ in the perioperative setting and even took other drugs such as xueshuantong injection and xuesaitong injection to improve microcirculation at the same time. Notably, some patients took both Lipo-PGE_1_ and hemostatic drugs after the operation, which would increase the risk of postoperative hemorrhage. Given this fact, we stipulate that if a patient takes Lipo-PGE_1_ 1 day before the operation or 2 days after the operation, this case should be considered as a contraindication.

During the study period, alprostadil injection (10 μg) and alprostadil dried emulsion for injection (5 μg) were available. The indications, dosage, administration route, contraindications, etc. specified in the instructions for the two drugs are identical. But since alprostadil injection (10 μg) is produced by Beijing Tide Pharmaceutical Co., Ltd., China and was first listed in China, it is cheaper. Moreover, there is no evidence of a difference in the safety and efficacy between the two drugs so far. In addition, the inserts clearly state that the dose of Lipo-PGE_1_ is 5–10 μg per day. Therefore, clinicians should prefer the cheaper drug unless this drug is insufficient or patients only need 5 μg Lipo-PGE_1_ per day.

A previous study showed the daily dose of Lipo-PGE_1_ was significantly different for different diseases and different hospitals, where 21.18% was more than 12.5 μg per day^15^. Another two studies reported that incidences of inappropriate doses were 34.43% and 39.68%, respectively^23,28^. In our results, 31 (11.57%) patients took Lipo-PGE_1_ with an inappropriate dose in the pre-intervention phase, yet all patients took Lipo-PGE_1_ with appropriate dose and frequency in the intervention and post-intervention phases. Nevertheless, the studies on high-dose use of Lipo-PGE_1_ are limited and the sample sizes in these studies were small^29,30^, which results in the lack of clear evidence-based medicine to support high-dose usage so far. Based on this, we suggest that Lipo-PGE_1_ should be used strictly following the dosage and frequency recommended by the summary of product characteristics in clinical practice.

Lipo-PGE_1_ is a formulation with lipid microspheres as the carrier of alprostadil, which is an O/W type sub-microemulsion. When superabundant hydrosoluble solvents are added to this formulation, the demulsification is easy to be caused, which would decrease its targeting effect and therapeutic efficacy. Moreover, demulsification may lead to the leakage of prostaglandin E1, an inflammatory factor and heat source, which stimulates blood vessels to produce serotonin and bradykinin, thus increasing capillary permeability and causing adverse reactions, such as phlebitis^31,32^. In our study, we found that Lipo-PGE_1_ was always diluted by large amounts of inappropriate solvents (0.02 or 0.04 or 0.1 μg/mL), such as invert sugar injection, multiple electrolytes, and invert sugar injection, which include fructose, unsuitable for patients with hyperuricemia and gout, and were expensive. During and after the intervention, the types and volumes of these solvents tended to be more reasonable. Unfortunately, volumes in all cases were still incorrect. Notably, since clinicians were reluctant to record them in medical records, the occurrences of adverse drug events (ADEs) in three phases of the intervention were not very clear and cannot be compared. Because of this fact, to improve the therapeutic effect and reduce the risk of ADEs, intravenous injection of Lipo-PGE_1_ should be strongly advocated.

Similar to most adjuvant drugs, the off-label use of Lipo-PGE_1_ was very serious. To improve the safety of patients and reduce the risk to medical staff, the standardized management of off-label use of Lipo-PGE_1_ is particularly important in the clinic. After simple administrative intervention, the improvement of the rationality of Lipo-PGE_1_ was still not satisfactory, which may be related to the following two reasons. On one hand, effective communication about the irrationality of Lipo-PGE_1_ usage between the leaders of clinical departments was lacking. On the other hand, clinicians did not pay enough attention to the off-label use of Lipo-PGE_1_, and many misunderstandings have been ingrained. In light of the above, the number and working hours of the clinical pharmacist should be increased, which would contribute to more extensive medication education, in-depth communication between surgeons and patients, and a superior doctor-patient relationship in the future.

Of course, our study has some limitations. First, this intervention study is a self-control study design, which misses a simultaneous control group, thus lacking enough persuasion than a prospective, controlled study design. Second, the data in this paper were only collected from the Affiliated Hospital of Southwest Medical University. It should be noted that, whereas data were obtained from one hospital, we believe these findings are generalizable to other hospitals in China based on the merits of our methodology as well as the hospital size and geographical location, the largest public hospital in the south of Sichuan Province. Third, we spent a whole year eliminating any potential effect of seasonality, but we did not identify any other potential factors that may influence the use of Lipo-PGE_1_, such as shortage of medicine and turnover of surgeons in this teaching hospital during the research period. Thus, these obtained favorable results could not be attributed solely to administrative intervention. Lastly, although significant reductions of inappropriate Lipo-PGE_1_ use in neurosurgery were observed during and after the intervention, the results were not satisfactory. We will take measures to further rectify the administration route and menstruum volume. Altogether, the reliability of administrative intervention needs to be confirmed in more rigorous studies in the future.

## Conclusions

In summary, the off-label use of Lipo-PGE_1_, as an adjuvant drug, is very common in neurosurgery. The simple administrative intervention reduced Lipo-PGE_1_ use and the reduction was maintained or even better in the 1-year post-intervention period, which contributing to favorable economic outcomes. However, there still remains a number of inappropriate uses of Lipo-PGE_1_. To further improve the rational use of Lipo-PGE_1_, a combination of administrative intervention and real-time clinical pharmacists intervention should be implemented.

## Data Availability

The datasets used and/or analyzed during the current study are available from the corresponding author on reasonable request.

## References

[1] Tondi P (2004). Treatment of ischemic ulcers of the lower limbs with alprostadil (prostaglandin E_1_). Dermatol. Surg..

[2] Zhang LP (2018). Alprostadil attenuates myocardial ischemia/reperfusion injury by promoting antioxidant activity and eNOS activation in rats. Acta Cir. Bras..

[3] Kolotylo A, Venher I, Kostiv S, Iftodiy A (2018). Endothelial dysfunction and microscirculation features in patients with high risk of development of reperfusion syndrome in conditions of reconstruction arterial operations. Georgian Med. News.

[4] Antonio LGM, Evora PRB, Piccinato CE (2009). Use of alprostadil, a stable prostaglandin E_1_ analogue, for the attenuation of rat skeletal muscle ischemia and reperfusion injury. Minerva Chir..

[5] Seo, H., Lopez, C. N., Succar, L. & Donahue, K. R. Evaluation of inhaled alprostadil in hospitalized adult patients. *Ann. Pharmacother.*10.1177/10600280211042675 (2021). 10.1177/10600280211042675 34486414

[6] Reddy R, Diaz P, Ramasamy R (2022). Re: Association of phosphodiesterase-5 inhibitors versus alprostadil with survival in men with coronary artery disease. Eur. Urol..

[7] Aykanat A (2015). Long-term prostaglandin E_1_ infusion for newborns with critical congenital heart disease. Pediatr. Cardiol..

[8] Han K, Liu C, Shi X, Rao X (2018). Effects of alprostadil combined with calcium dobesilate in patients with diabetic peripheral neuropathy. Neuro Endocrinol. Lett..

[9] Machado-Alba, J. E. & Machado-Duque, M. E. Use of intravenous alprostadil in patients with severe critical ischemia of the lower limbs. *Vascular* **27**, 318–323. 10.1177/1708538118816267 (2019). 10.1177/1708538118816267 30563434

[10] Sheng XC (2018). Intracoronary infusion of alprostadil and nitroglycerin with targeted perfusion microcatheter in STEMI patients with coronary slow flow phenomenon. Int. J. Cardiol..

[11] Wang JW (2021). Lipid microsphere-coated PGE1 improves peritoneal transport and reduces inflammation in peritoneal dialysis: A randomized clinical pilot trial. Semin. Dial..

[12] Li JY (2020). Multiparametric MRI evaluation of liposomal prostaglandins E_1_ intervention on hepatic warm ischemia-reperfusion injury in rabbits. J. Magn. Reson. Imaging.

[13] Jin SJ (2019). Lipo-prostaglandin E_1_ increases immediate arterial maximal flow velocity of free flap in patients undergoing reconstructive surgery. Acta Anaesthesiol. Scand..

[14] Yu CQ (2002). Characteristics of preparation of Lipo-PGE_1_. Chin. Hosp. Pharm. J..

[15] Wang H, Liu ZY, Ding Y, Yang ZW (2017). Analysis of clinical features of alprostadil lipid-micro injection from 159 hospitals of China. China Pharmacy.

[16] Landwehr C (2019). Cross-sectional survey of off-label and unlicensed prescribing for inpatients at a paediatric teaching hospital in Western Australia. PLoS ONE.

[17] Cui J, Zhao L, Liu XH, Liu MYJ, Zhong LH (2021). Analysis of the potential inappropriate use of medications in pediatric outpatients in China. BMC Health Serv. Res..

[18] Kruger M (2019). Off-label use in ambulatory paediatric clinics in a central South African hospital. J. Trop. Pediatr..

[19] Magalhaes J (2015). Use of off-label and unlicenced drugs in hospitalised paediatric patients: A systematic review. Eur. J. Clin. Pharmacol..

[20] Carton L (2015). Off-label prescribing of antipsychotics in adults, children and elderly individuals: A systematic review of recent prescription trends. Curr. Pharm. Des..

[21] Zhang LL (2012). Evidence-based evaluation on off-label drug use policies in 15 countries. Chin. J. Evid-based. Med..

[22] Cao AL, Tang YL, Qian J, Wang Z (2018). Interventional analysis on the off-label drug use of alprostadil lipid microsphere injection. J. Pharm. Pract..

[23] Lin L, Lao HY, Lun YN, Li YP, Yang M (2016). Interventional effect of off-label drug use of alprostadil injection among inpatients in Guangdong general hospital: A before-after study. Chin. J. Evid-based. Med..

[24] Luo HL, Fan QZ, Xiao SL, Chen K (2017). Impact of clinical pharmacist interventions on inappropriate prophylactic acid suppressant use in hepatobiliary surgical patients undergoing elective operations. PLoS ONE.

[25] Society of Infectious Diseases, CMA, Chinese Society of Hepatology, CMA. Guidelines for the diagnosis and treatment of liver failure. *J. Pract. Hepatol.* **16**, 210–216 (2013).

[26] American College of Cardiology Foundation, American Heart Association. Management of patients with peripheral artery disease (compilation of 2005 and 2011 ACCF/AHA guideline recommendations): a report of the American College of Cardiology Foundation/American Heart Association Task Force on practice guidelines. *J. Am. Coll. Cardiol.* **61**, 1555–1570. 10.1016/j.jacc.2013.01.004 (2013). 10.1016/j.jacc.2013.01.004 PMC4492473 23473760

[27] Yang SC, Wang JM, Chen LJ, Wang SJ, Hong WY (2015). Evidence based research on the off-label uses of alprostadil injection in our hospital. Prac. Pharm. Clin. Remed..

[28] Lin L, Lao HY, Lun YN, Yang M (2015). Application of PDCA cycle management in the intervention of off-label dosage of alprostadil injection. China Pharm..

[29] Hong LH, Zhang J, Shen JG (2015). Clinical efficacy of different doses of lipo-prostaglandin E_1_ in the treatment of painful diabetic peripheral neuropathy. J. Diabetes Complications.

[30] Guan, H. L. *et al*. The clinical effectiveness and safety of alprostadil combined with alpha lipoic acid in the treatment of diabetic peripheral neuropathy: A protocol for systematic review and meta-analysis. *Medicine (Baltimore)* **99**, e23507. 10.1097/md.0000000000023507 (2020). 10.1097/MD.0000000000023507 PMC7738071 33327292

[31] Yamauchi K, Yasunaga S, Kawasaki H, Kurosaki Y (2003). An approach to predict the ductus-arteriosus dilating effect induced by lipo-prostaglandin E_1_ in newborn rats lacking plasma concentration-time data by the pharmacological response kinetic model. Biol. Pharm. Bull..

[32] Iwaki R (2020). Effect of long-term administration of prostaglandin E_1_ on morphologic changes in ductus arteriosus. Ann. Thorac. Surg..

